# Missed Evaporation from Atmospherically Relevant Inorganic Mixtures Confounds Experimental Aerosol Studies

**DOI:** 10.1021/acs.est.2c06545

**Published:** 2023-02-09

**Authors:** Jenny Rissler, Calle Preger, Axel C. Eriksson, Jack J. Lin, Nønne L. Prisle, Birgitta Svenningsson

**Affiliations:** † Ergonomics and Aerosol Technology, Lund University, Box 118, 221 00 Lund, Sweden; ‡ Bioeconomy and Health, Research Institutes of Sweden (RISE), Scheelevägen 17, 223 70 Lund, Sweden; § MAX IV Laboratory, Lund University, Box 118, 221 00 Lund, Sweden; ∥ Center for Atmospheric Research, University of Oulu, P.O. Box 4500, 90014 Oulu, Finland; ⊥ Physics Department, Lund University, Box 118, 221 00 Lund, Sweden

**Keywords:** sea spray, sea salt, inorganic aerosol mixtures, hygroscopicity, thermodynamics

## Abstract

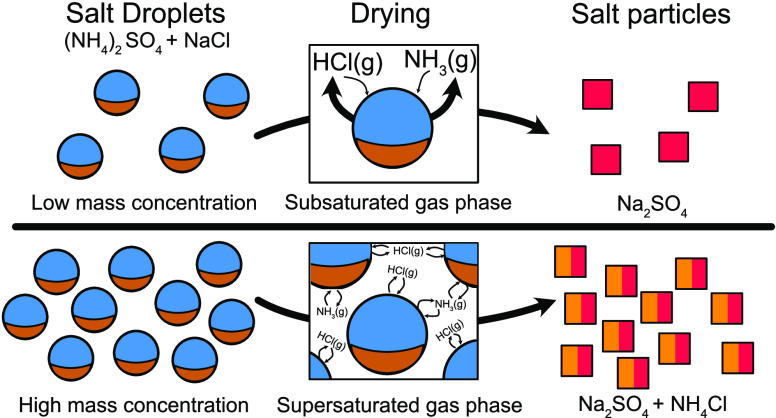

Sea salt aerosol particles are highly abundant in the atmosphere and play important roles in the global radiative balance. After influence from continental air, they are typically composed of Na^+^, Cl^–^, NH_4_^+^, and SO_4_^2–^ and organics. Analogous particle systems are often studied in laboratory settings by atomizing and drying particles from a solution. Here, we present evidence that such laboratory studies may be consistently biased in that they neglect losses of solutes to the gas phase. We present experimental evidence from a hygroscopic tandem differential mobility analyzer and an aerosol mass spectrometer, further supported by thermodynamic modeling. We show that, at normally prevailing laboratory aerosol mass concentrations, for mixtures of NaCl and (NH_4_)_2_SO_4_, a significant portion of the Cl^–^ and NH_4_^+^ ions are lost to the gas phase, in some cases, leaving mainly Na_2_SO_4_ in the dry particles. Not considering losses of solutes to the gas phase during experimental studies will likely result in misinterpretation of the data. One example of such data is that from particle water uptake experiments. This may bias the explanatory models constructed from the data and introduce errors inte predictions made by air quality or climate models.

## Abstract 2

Experimental data on mixtures of the four most abundant inorganic ions in the atmosphere can be misinterpreted if the evaporation of HCl (g) and NH_3 _(g) is not considered.

## 1 Introduction

The interactions between aerosol particles and water vapor play key roles in the atmosphere. Their fates are intricately connected and influence air quality,^1^ atmospheric chemistry,^2^ and climate.^3^ Based on aerosol flux and turnover of global water vapor and cloud water, it is estimated that water vapor, on average, condenses and evaporates 10 times before finally precipitating and that, on average, each aerosol particle has been cycled through the global cloud system three times.^4^ Cloud and fog waters are both sources and sinks of particulate matter in the atmosphere.^5^ The dissolution of gases to the aqueous phase can lead to a wide range of chemical reactions resulting in the formation of secondary aerosol mass that would not have occurred in the gas phase.^6^ The cloud processing contributes to atmospheric aerosol particle mass by the same order of magnitude as the release of particle mass at the surface.^4^

The adsorption of water by aerosol particles and the formation of an aqueous particle or droplet phase is governed by the ambient relative humidity (RH) and the hygroscopicity of the aerosol particle. Atmospheric aerosol particles are typically composed of mixtures of inorganic and organic compounds in various fractions depending on the location.^7^ The hygroscopicity of inorganic compounds is generally considered well understood and often adequately described using parameterizations of water activity such as the Zdanovskii–Stokes–Robinson (ZSR) mixing rule^8−10^ or *κ*-Köhler theory.^11−13^ In fact, inorganic salts, in particular ammonium sulfate and sodium chloride, are widely used as reference compounds for the calibration of instruments that measure aerosol hygroscopicity, such as the hygroscopic tandem differential mobility analyzer^14^ (HTDMA), the differential aerosol sizing and hygroscopicity spectrometer probe^15^ (DASH-SP), and the cloud condensation nuclei counter^16^ (CCNC). Deviations from theoretical predictions of aerosol hygroscopic behavior of mixtures of inorganic and organic mixtures have generally been attributed to the presence of the organic aerosol compounds giving rise to aqueous phase nonidealities, such as the partial dissolution of certain organic species with finite water solubility,^17−19^ aqueous surface tension depression,^20−22^ bulk/surface partitioning,^23−27^ or formation of micelles and other self-assembled structures.^28,29^ However, experimental studies have again brought into question the ability to accurately predict the hygroscopic behavior of inorganic compounds in mixtures based solely on their pure-component properties. In a study of purely inorganic model sea salt particles, using an HTDMA and an electrodynamic balance, Zieger and co-authors found the hygroscopicity of sea salt particles to be lower than that of pure NaCl.^30^ The reduction in hygroscopicity was hypothesized to be due to the presence of hydrates. In another study, it was shown that the observed deliquescence behavior of internally mixed particles composed of NaCl/(NH_4_)_2_SO_4_ deviates from that predicted by a thermodynamic model when in mole fractions varying from 0.40 to 0.77.^31^ Furthermore, experiments using synchrotron radiation-based X-ray photoelectron spectroscopy (XPS) has also found segregation of inorganic ions in aerosol particles.^32,100^

## 2 Background and Formulating the Hypothesis

In the earlier work by Svenningsson and co-authors,^33^ the hygroscopic growth of particles of four atmospherically relevant mixtures of inorganic/organic compounds was studied. Mixtures included three inorganic salts (ammonium sulfate, ammonium nitrate, and sodium chloride) and three model organic compounds (levoglucosan, succinic acid, and fulvic acid). The mixtures were studied at both subsaturation (RH 30–95%) using an HTDMA and at supersaturation using a CCNC. The ZSR mixing rule was used to predict water uptake of the mixtures based on that of the pure compounds, which was found to adequately explain the hygroscopic growth for three out of the four mixtures at both sub- and supersaturation. For the mixture representing sea salt particles, MIXSEA, based on the composition suggested by Raes and co-authors^34^ (more details found in the Supporting Information), the measured water uptake was significantly lower than that predicted by the ZSR mixing rule, both at ambient water vapor subsaturation and at supersaturation, and the shape of the hygroscopic growth factor curve as a function of RH was poorly described using the ZSR mixing rule (Figure S1a). MIXSEA was the only mixture containing both NaCl and (NH_4_)_2_SO_4_, while the organic components in the mixture were succinic acid and fulvic acid. In the paper where the results were first presented, the presence of organics and their effect on surface tension was the main hypothesis to explain these observed deviations. However, a considerable reduction in surface tension would be required for it to be the sole cause of the observed deviation between the ZSR mixing rule and experimental results. Furthermore, the critical water supersaturation needed to activate the particles to cloud droplets could be relatively well predicted from the hygroscopic growth factors (HGFs) measured at subsaturation by applying basic Köhler theory.^13^ Since a reduction in droplet surface tension will affect the water uptake differently at the subsaturated and at the supersaturated conditions required for cloud droplet activation (i.e., the critical supersaturation),^13,35^ it is unlikely that the critical supersaturation needed for CCN activation would be correctly predicted from the HGFs at subsaturation if the surface tension was strongly affected by the organics.

To explain the observed deviations between theory and experiments, we identified and evaluated several alternative hypotheses. These include the particle shape factor and effective density, the formation of hydrates, and lastly, that known thermodynamics of aqueous solutions including both NaCl and (NH_4_)_2_SO_4_ are not fully accounted for in experimental work on aerosols formed by drying nebulized solutions. Our latter hypothesis was found to best explain the earlier reported experimental observations, as described in more detail in the Supporting Information.

More specifically, this hypothesis is that during the drying of nebulized droplet solutions of mixtures containing both NaCl and (NH_4_)_2_SO_4_, such as sea salt particles after influence from continental air, inorganic ions recombine to form molecular HCl and NH_3_, which subsequently evaporate from the aerosol phase. The resulting change in the composition of the dry aerosol particles may be of crucial importance for the interpretation of all laboratory studies of mixtures containing both NaCl and (NH_4_)_2_SO_4_, affecting particle properties such as water uptake. In this paper, we present experimental evidence, including HTDMA measurements and aerosol mass spectrometry, to substantiate our hypothesis. The experimental evidence is complemented by thermodynamic modeling, demonstrating the effect at realistic and often prevailing experimental and ambient aerosol mass concentrations. The other hypotheses initially investigated are described in the Supporting Information.

**Figure 1:**
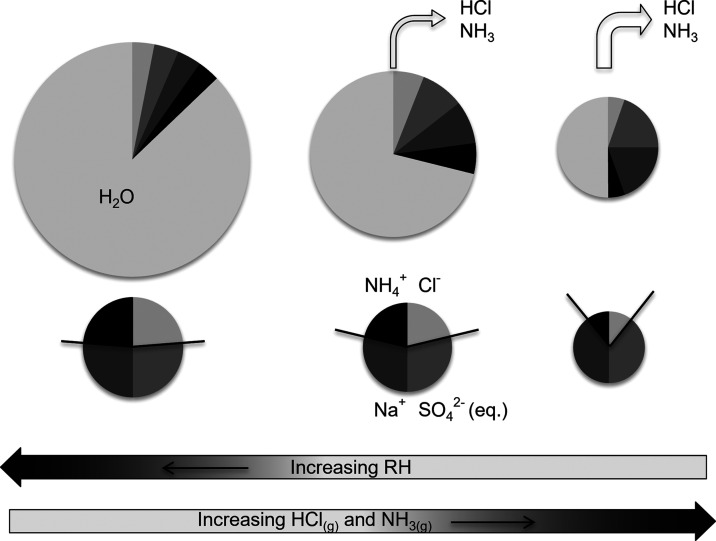
Schematic illustration of the evaporation of HCl and NH_3_ during drying of the mixed NaCl and (NH_4_)_2_SO_4_ droplet. The top row illustrates the change in droplet composition as RH is decreased, resulting in the evaporation of water, HCl, and NH_3_. The second row illustrates the composition of the solute. For both rows, the size of the circles qualitatively reflects the remaining water and the number of soluble ions.

The drying of the aerosol is a key experimental step performed in nearly all laboratory studies of particle water uptake. As the nebulized droplet dries when subjected to decreasing RH, the solution becomes more concentrated and the equilibrium state for Cl^–^ and NH_4_^+^ between the particle phase and gas phase are shifted, leading to the evaporation of HCl (g) and NH_3_ (g). The principle is illustrated in Figure 1, where the top row illustrates the change in the chemical composition of the droplets as they are exposed to decreasing RH (going from left to right in the figure), and the second row illustrates the relative composition of the solutes as HCl (g) and NH_3 _(g) leave the droplet.

## 3 Experimental Methods

The composition of the aerosol particles studied was a mixture of NaCl and (NH_4_)_2_SO_4_ in molar ratios 2:1, resulting in an ion molar ratio of 1:1:1:0.5 (Na^+^/Cl^–^/NH_4_^+^/SO_4_^2–^). This specific molar ratio was used since, in theory, it could result in the formation of pure Na_2_SO_4_ and NH_4_Cl in the dry state (predicted by the E-AIM model to be formed upon crystallization preferred over NaCl and (NH_4_)_2_SO_4_), with potentially the maximal evaporation of HCl and NH_3_. For more details, see the Supporting Information, where the results from modeling the evaporation at molar ratios of NaCl to (NH_4_)_2_SO_4_ from 0:1 and 1:0 are shown (Figure S2).

To test the hypothesis, two types of experiments were performed:

(1) Hygroscopic growth factor (HGF) measurements as a function of RH at water subsaturation (*a*_w_ < 1) using an HTDMA setup.
(2) Measurements of the chemical composition of the particles using the aerosol mass spectrometer (AMS).

In both types of experiments, the aerosol particles were generated from prepared solutions using a collision-type nebulizer and the generated droplets were dried using diffusion driers. During the AMS [Aerodyne Research Inc.] experiments, the mass/volume concentrations of the particles needed to predict the evaporation dynamics were estimated from the mobility number size distributions measured with a scanning mobility particle sizer (SMPS), TSI 3082.

The HTDMA measures the diameter increase of dry particles due to hygroscopic growth when exposed to a specific relative humidity. The HGF is defined as the ratio between the wet and dry mobility diameter of the particle. The experimental setup is described in detail by Svenningsson and co-authors,^33^ where details about the calibration procedure and data quality assurance are included. The HTDMA can be operated in different modes: scanning RH for particles of one dry size or varying the dry size at a fixed RH. During the current measurements, the RH scanning mode was used, scanning RH from 20 to 98%. RH is related to water activity (*a*_w_) as *a*_w_ = RH/100/*C*_Ke_, where *C*_Ke_ is the Kelvin curvature correction factor.^36^ Both the deliquescence branch (going from dry to wet state) and the efflorescence branch (going from wet to dry state) were studied.

An AMS^37^ was used to investigate the particles’ composition under varied particle mass concentrations. To investigate if and how the particle composition changed with changing aerosol mass concentration, the ratio of the fragments NH_3_^+^ to SO_2_^+^ detected with the AMS was used, probing the relative mass ratio of NH_4_^+^ to SO_4_^2–^ in the particles. The normal approach for AMS data analysis, which is to add up all ions contributing to each species for quantification, was not feasible because the AMS detection scheme relies on flash vaporization. This may result in artifacts due to the unwanted production of thermal ions and incomplete vaporization of “refractory” components, such as Na_2_SO_4_, and other unwanted particle–vaporizer interactions including chemical reactions between the vaporizer and particle components such as NaCl.^38^ Different aerosol mass concentrations were achieved by diluting the nebulizer solution stepwise from 1 to 0.025 g/L while keeping all other settings used for nebulization and drying fixed.

## 4 Thermodynamic Modeling Using E-AIM

For thermodynamic modeling, the E-AIM model was used (http://www.aim.env.uea.ac.uk/aim/aim.php Model III).^39^ In this work, the model was used to evaluate

(1) the compounds formed in the dry particles (case 1),
(2) the hygroscopic growth and deliquescence behavior of the particles (case 2), and
(3) the gas phase/aqueous phase partitioning (case 3).

All of the modeling was done for 298 K. Below, we provide details on the various simulation conditions used in each specific simulation set (cases 1–3 in Table 1). In the modeling, no effect of surface tension or other properties related to the particle size such as bulk/surface partitioning was considered.

**Table 1:** E-AIM Modeling Settings

process modeled	case	composition, molar ratios Na^+^(Cl^–^)/NH_4_^+^(SO_4_^2–^)	conc. [mol m^–3^]	*a*_w_	gas phase allowed	crystals allowed	aq. density
crystals	1	1(1):1(0.5)	NA^c^	0.50	N	Y^a^	
HGFs and deliquescence	2a	1(1):1(0.5)	NA^c^	0.30–0.95	N	Y^a^	vol. add.^b^
	2b	50(1):1(25)	NA^c^	0.30–0.95	N	Y^a^	vol. add.^b^
	2c	50(1):1(25)	NA^c^	0.30–0.95	N	Y^a^	E-AIM
gas/particle partitioning	3a	1(1):1(0.5)	10^–7^, 10^–6^, 10^–5^	0.10–0.95	Y	N	
	3b	1(1):1(0.5)	10^–8^–10^–4^	0.30, 0.50	Y	N	

a Crystals allowed: NaCl, Na_2_SO_4_, (NH_4_)_2_SO_4_, and NH_4_Cl.

b Volume additivity was assumed for the solute/water in the droplets.

c Input concentrations, in this case, do not impact the model output and are therefore not provided.

The aim of the calculations made using the settings in case 1 was to address the question of what salt compounds are preferentially formed after drying the droplet solution, assuming no loss of solutes to the gas phase. Thus, in case 1, any evaporation of the solute into the gas phase was inhibited. In the simulation, (NH_4_)_2_SO_4_, NaCl, Na_2_SO_4_, and NH_4_Cl were allowed to form, while Na_2_SO_4_·(NH_4_)_2_SO_4_·4H_2_O and Na_2_SO_4_·10 H_2_O were not. Excluding the salts with crystal water was motivated by crystallization in sub-micrometer droplets, which often form crystals without water, and the initial test showed that these hydrates were present only in a very narrow range of *a*_w_ (0.68–0.73), which is below the *a*_w_ of the modeled deliquescence point and above that where crystallization occurs. The simulation was performed at *a*_w_ 0.5 to simulate a case with dry particles without an aqueous phase.

In case 2 (Table 1), the HGFs were calculated. The number of moles of water associated with a given composition of the solute composition was calculated as a function of *a*_w_ assuming spherical particles (i.e., having a dynamic shape factor of 1). The density of the aqueous droplets was determined by either assuming volume additivity (cases 2a and 2b in Table 1) or using the density of the solutions given by the E-AIM model (case 2c). First, the deliquescence and HGFs were modeled for the mixture according to the ion molar ratios of the solutes as in the nebulized solution—as if no evaporation of the solutes occurs (case 2a in Table 1). Evaporation was not treated explicitly in the E-AIM model. Instead, the effect of the loss of ions to evaporation on the point of deliquescence was simulated by repeating the calculations, removing equal amounts of NH_4_^+^ and Cl^–^ successively.

For comparison, the HGFs of Na_2_SO_4_ were estimated from electrodynamic balance (EDB) data.^40^ Since the EBD data provides particle hygroscopic mass growth factors, while the HTDMA gives diameter growth factors, an assumption of densities of the dry and wet particles is needed to translate the mass increase to diameter growth. See further description in Section 5.1.

Lastly, the E-AIM model was used to evaluate the gas/particle phase ion partitioning (case 3 in Table 1). In this case, the aerosol mass concentrations (in moles solute per cubic meter of air) in a range relevant to atmospheric and laboratory conditions were inserted in the model: 10^–7^, 10^–6^, and 10^–5^ mol equiv/m^3^ of each ion. Here, 1 mol equiv refers to the moles of the respective ion needed to react with 1 mol of electrons or hydrogen ions, i.e., 1 mol of Na^+^, NH_4_^+^, and Cl^–^ and 0.5 mol of SO_4_^2–^. As an example, for sulfate (with a molar mass of 96.06 g/mol), these molar equivalents correspond to aerosol mass concentrations of ∼5, 50, and 500 μg/m^3^, respectively.

To model the efflorescence branch of the hygroscopic growth curve, the model was set to not allow any crystal structures. The efflorescence branch corresponds to supersaturated solutions being formed during the drying of the solution droplets.

## 5 Results and Discussion

For solutions with Na^+^, Cl^–^, NH_4_^+^, and SO_4_^2–^ in molar ratios of 1:1:1:0.5, the E-AIM model predicts that upon crystallization, the ions combine primarily to Na_2_SO_4_ and NH_4_Cl (case 1).

### 5.1 Particle Hygroscopic Growth and Deliquescence of Inorganic Salt Mixtures

**Figure 2:**
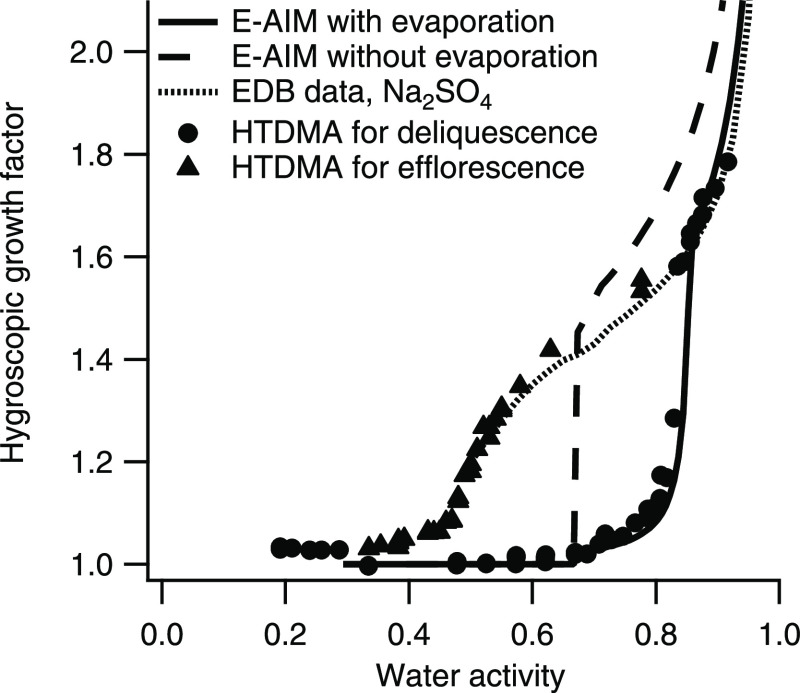
HGFs for the inorganic mixture (Na^+^, Cl^–^, NH_4_^+^, SO_4_^2–^ in molar ratios 1:1:1:0.5) measured by the HTDMA for the deliquescence branch (circles) and the efflorescence branch (triangles), and the HGFs curves of the deliquescence branch modeled using the E-AIM model (model settings as in cases 2a and 2b in Table 1): either assuming no evaporation (molar proportion 1:1:1:0.5 of the solute ions), corresponding to the dashed line, or assuming nearly full evaporation of Cl^–^ and NH_4_^+^ (resulting in molar proportions 50:1:1:25), corresponding to the solid line. The HGF estimated from EDB data of pure Na_2_SO_4_ is also shown (dotted line).

To test the hypothesis described in Section 2, HGFs were modeled over *a*_w_ (∼0.20 to 0.98) using E-AIM and compared to those measured using the HTDMA. The experimentally determined HGF (*a*_w_) curve of the salt solutions prepared, nebulized, and dried (i.e., Na^+^, Cl^–^, NH_4_^+^, SO_4_^2–^ in molar ratios 1:1:1:0.5) is shown in Figure 2, including both the deliquescence branch and the efflorescence branch. The HGF curves are also shown in the Supporting Information together with the corresponding HGF curves for a mixture representing sea salt (MIXSEA).^33^

The deliquescence HGF (*a*_w_) curve for the inorganic mixture was modeled using the settings given for case 2a (Table 1). When compared to the experimentally determined HGF curves, the modeled water uptake exceeds the measured water uptake. However, the most pronounced difference between measured and modeled data is seen in *a*_w_ at deliquescence.

To simulate the HGF (*a*_w_) for a solution where ions are lost to the gas phase by the evaporation of HCl (g) and NH_3_ (g), the growth curves were modeled for solutions successively removing NH_4_^+^ and Cl^–^ in equal proportions. In Figure 2 (black solid line), one example of these results is shown: the predicted deliquescence and HGF curve for a mixture with molar proportions of 50:1:1:25 (settings/conditions given as case 2b in Table 1). This specific example was selected as it is in good agreement (compare in Figure 2) with the experimental HGF (*a*_w_) data, both with respect to HGFs and *a*_w_ at deliquescence and corresponds to nearly all Cl^–^ and NH_4_^+^ being lost from the particle phase (only ∼2% remaining). The amount of Cl^–^ and NH_4_^+^ remaining in the particle phase mainly influences the tail of low HGF values for *a*_w_ just below the “main” deliquescence (*a*_w_ in the range 0.7–0.8). The HGF of the same mixture was also modeled using the density of the solution according to E-AIM, resulting in nearly overlapping HGF when assuming volume additivity (as defined in model 2c in Table 1), not shown in Figure 2.

Under the assumption that HCl and NH_3_ are fully evaporated, the dry aerosol particles will crystallize to form Na_2_SO_4_. Therefore, as a second control, the measured HGFs were compared to those of Na_2_SO_4_, predicted from a parameterization based on EDB data^40^ using a density of the dry particle of 1730 g/m^3^ to translate the mass-based water uptake from EDB data into diameter growth (HGFs). This density is based on the fitting of the HGFs from EDB data to HGFs measured by the HTDMA of pure Na_2_SO_4_ particles at 0.90 *a*_w_ for particles generated under the same conditions (same nebulizer and drying conditions) as during the measurement of HGFs of the salt mixture. This effective density includes crystal density, nonsphericity/porosities, and potentially, also hydrates in the crystalline particles, further elaborated on in the Supporting Information. The shape of the measured HGFs as a function of *a*_w_ is well described by that of pure Na_2_SO_4_.

Taken together, the comparison between modeled and measured HGFs strongly indicates that, under the specific experimental conditions, the evaporation of HCl and NH_3_ from the aerosol phase is nearly complete, resulting in nearly pure Na_2_SO_4_ particles after nebulizing and drying the 2:1 NaCl/(NH_4_)_2_SO_4_ salt mixture.

MIXSEA revealed a stronger shift in the HGF between the case when assuming no evaporation and the case assuming the full evaporation of HCl (g) and NH_3_ (g) from the particles (Figure S1). An explanation for this is that the evaporation of the inorganic ions results in particles composed of a larger fraction of organics with low HGFs. In Figure S1, we show that when assuming the full evaporation of Cl^–^ and NH_4_^+^ from the particles, the modeled and measured HGFs agree well, and thus the deviation observed between modeled and measured HGFs presented in the study by Svenningsson and co-authors^33^ is likely not explained by the organics, but involves the same processes related to the inorganic salts solely, as described here.

The hygroscopic properties of mixtures of inorganic salts were studied in a work by Cohen and co-authors,^41^ including a mixture of NaCl and (NH_4_)_2_SO_4_. In their study, the hygroscopic properties were studied using an electrodynamic balance (EDB), determining the relative mass growth of a droplet as a function of water activity. The principle and measurement procedure is different from that of the HTDMA and CCNC. For example, in contrast to HTDMA or CCNC, the EDB works with super micron particles (aerodynamic diameters 11–21 μm). Consequently, the surface area to mass ratio of the droplet studied is different, resulting in a lower mass evaporation rate. Furthermore, the residence time before and during a measurement is in a different time range. Thus, the effect of losses to the gas phase might not be as large during EDB measurements as for techniques that study the water uptake of sub-micrometer particles generated by nebulizing and drying solutions. Nevertheless, Cohen and co-authors noted a small loss of the particle mass (2%) during a cycle (going from dry particle to high water activity and back to the dry state) for all mixtures containing (NH_4_)_2_SO_4_ and NaCl and mentioned the possibility of the evaporation of HCl (g) and NH_3_ (g) as a possible explanation. Furthermore, they report a deviation from the ZSR mixing rule for the mixtures that they hypothesize could be attributed to the dry particles of the mixture still containing some water (while completely dry for the pure salts). However, both these observations may hypothetically be explained by the loss of solutes to the gas phase through the evaporation of HCl (g) and NH_3_ (g).

### 5.2 Modeling the Partitioning Between the Gas/Particle Phase

The gas/particle partitioning was modeled at three aerosol concentrations: 10^–7^, 10^–6^, and 10^–5^ mol equiv/m^3^ as a function of *a*_w_ (*a*_w_ from 0.1 to 0.95). The prerequisite corresponds to conditions given as those of case 3a in Table 1. As a guidance, the aerosol mass concentrations corresponding to 10^–7^ mol equiv/m^3^ are for SO_4_^2–^ (50 nmol/m^3^) ∼5 μg/m^3^ and for Na^+^ (100 nmol/m^3^) ∼2 μg/m^3^.

**Figure 3:**
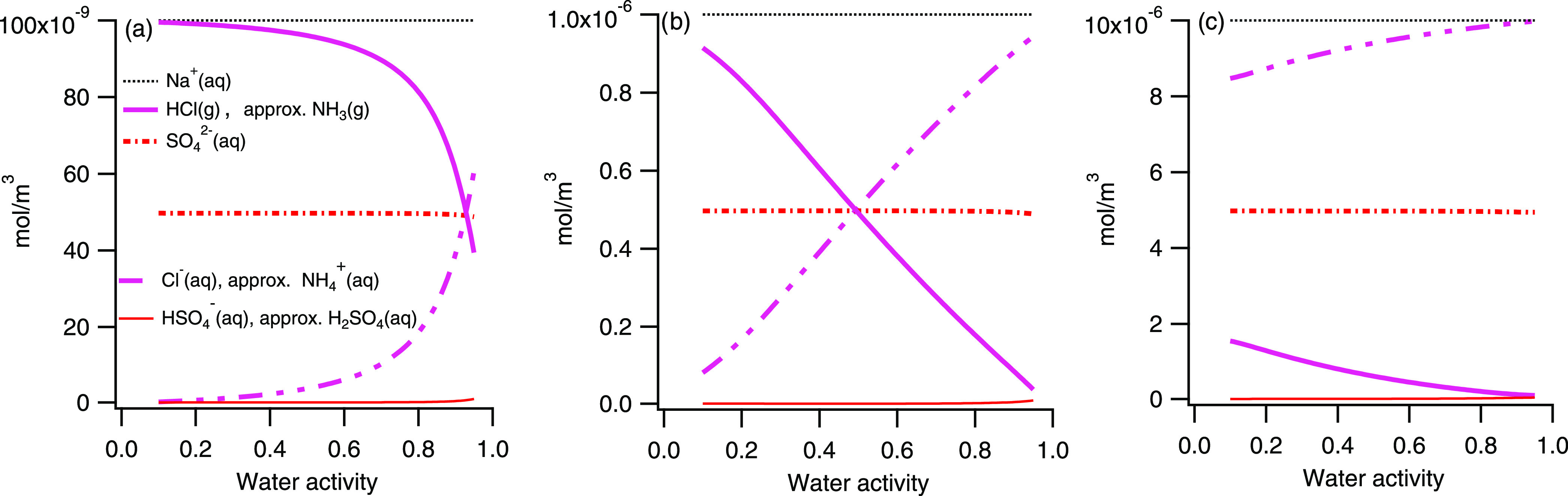
Gas/particle phase partitioning was modeled at 3 mol equiv concentrations in the air of Na^+^, Cl^–^, NH_4_^+^, and SO_4_^2–^ in molar ratios 1:1:1:0.5: 10^–7^ mol/m^3^ (a), 10^–6^ mol/m^3^ (b), and 10^–5^ mol/m^3^ (c) (for Na corresponding to a mass concentration of ∼2, 0.2 and 0.02 μg/m^3^, respectively) as a function of water activity.

The result shows the evaporation of HCl (g) and NH_3_ (g) from the particles at all three aerosol mass concentrations modeled. The evaporation becomes more pronounced the lower the concentration is and the lower the water activity is (see Figure 3). At the point of crystallization, which is assumed to take place at *a*_w_ ∼0.4 to 0.5, the evaporation is nearly complete at mole equivalent aerosol concentrations of ∼10^–7^ (Figure 3a). For the highest aerosol concentrations modeled, 10^–5^ mol equiv/m^3^ (10 μmol/m^3^), the effect of evaporation is still noticeable (Figure 3c) but minor. According to the model, SO_4_^2–^ and Na^+^ stay in the droplet at all concentrations. This means that during an experiment, the resulting chemistry of the generated particles can be significantly different, depending on the mass concentration of the solutes in the air during drying. The resulting aerosol mass concentration during drying of the droplet affects the equilibrium partitioning between the gas/particle phase of certain ions (i.e., Cl^–^ and NH_4_^+^) and thus the relative amount of the ion entities (i.e., Na^+^/Cl^–^/NH_4_^+^/SO_4_^2^) remaining in the particles after drying.

The respective evaporation of HCl and NH_3_ from the particles is in approximately the same amounts, i.e., they cannot be distinguished in Figure 3. There is, however, a small difference, resulting in slightly higher concentrations of H^+^ compared to OH^–^ in the aqueous phase. Still, the H^+^ concentration is at least a factor of 100 lower than the Na^+^ concentration. As shown in Figure 3, there is also a small fraction of the sulfate existing as HSO_4_ and H_2_SO_4_ in the liquid phase.

### 5.3 Particle Chemical Composition: Experimental vs. Modeled Results

As a final attempt to explore our hypothesis, the ion content of NH_4_^+^ and SO_4_^2–^ in the dried particles was measured using the AMS while varying the mass concentration in air. The concentration in air was varied by varying the concentration of the nebulized solution from 0.025 to 1 g/L, while keeping all other variables related to the nebulization and drying constant. A higher dry aerosol mass concentration corresponds to a higher salt concentration in the nebulized solution.

**Figure 4:**
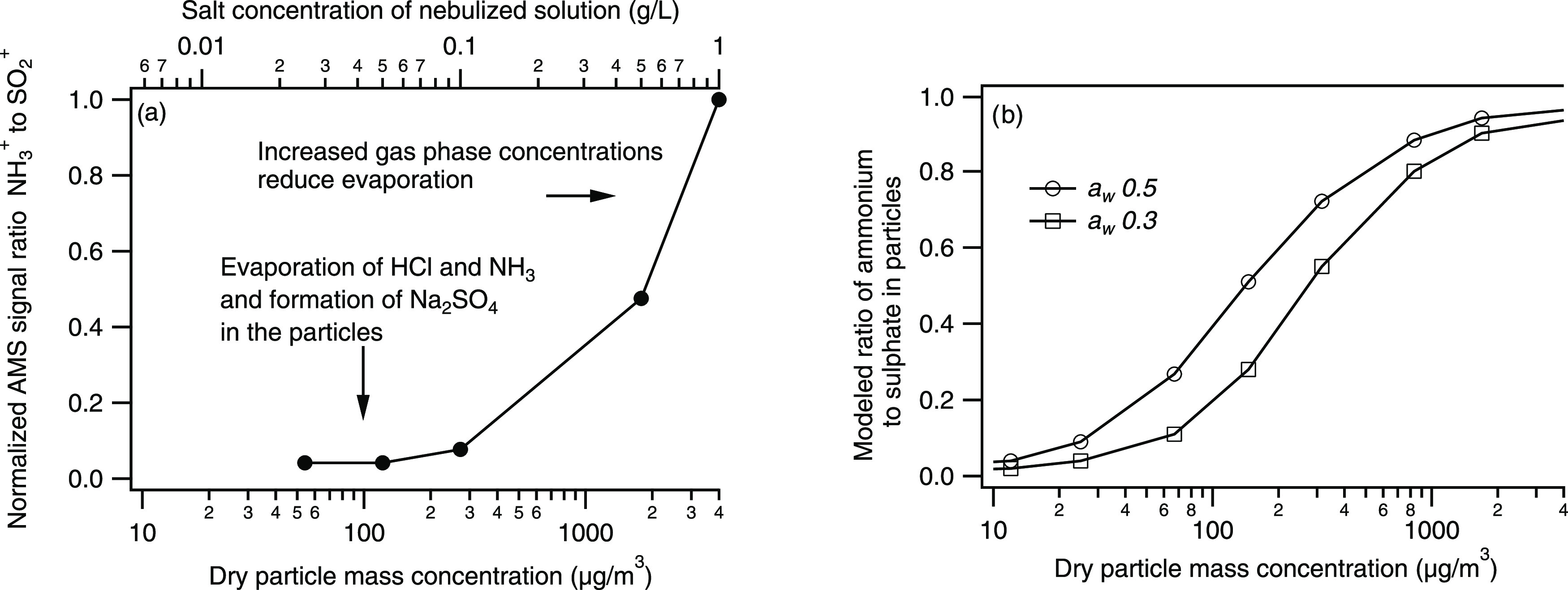
(a) AMS normalized signal ratio of NH_3_^+^ to SO_2_^+^ as a function of mass concentration estimated from the measured dry number size distribution resulting from nebulizing the 2:1 NaCl/(NH_4_)_2_SO_4_ solutions. The mass concentration is varied by changing the concentration of the nebulized solution, also shown in the top *x*-axis. (b) Modeled ratio between NH_4_^+^ and SO_4_^2–^ in the particle phase (E-AIM model) at various particle mass concentrations, modeled at an *a*_w_ of 0.30 (squares) and 0.5 (circles).

A first experimental observation was that adding the mass spectra for each pure salt nebulized separately (NaCl and (NH_4_)_2_SO_4_) did not yield the same mass spectrum as obtained from the mixed inorganic salt solutions (see Figure S4). Furthermore, the relative intensity of the peaks in the mass spectra varied with the concentration for the nebulized salt solution of the mixtures, even though the salts were added in the same proportions. When plotting the normalized molar ratios of NH_4_^+^ to SO_4_^2–^ (proxied by NH_3_^+^ and SO_2_^+^ measured by the AMS) as a function of concentration, the molar ratio decreases dramatically when decreasing dry aerosol mass concentration (Figure 4, left panel). Aerosol mass concertation was estimated from the measured dry particle number size distribution (mobility diameter), assuming spherical particles with a dry density of 2 g/cm^3^ (roughly approximated from the density of NaCl: 2.17 g/cm^3^, Na_2_SO_4_: 1.73-2.66 g/cm^3^, and (NH_4_)_2_SO_4_: 1.77 g/cm^3^).

The ratio of NH_4_^+^ to SO_4_^2–^ ions in the aerosol particle phase was modeled using E-AIM at similar mass concentrations as those estimated for the AMS experiment at an *a*_w_ of 0.3 and 0.5. The result is shown in the right panel of Figure 4 (model settings given in 3b in Table 1). In this case, we model the gas/particle phase partitioning with varying aerosol mass concentrations while keeping *a*_w_ constant. This is opposed to what is done in case 3a (Section 5.2), where we show partitioning as a function of *a*_w_, keeping the aerosol mass concentration constant (for each panel in Figure 3). The modeling revealed the same trend as observed in the experimental data, with decreasing NH_4_^+^ to SO_4_^2–^ molar ratios with decreasing aerosol particle mass concentration. This is again explained by the partial pressure of HCl (g) and HN_3_ (g) suppressing further evaporation of HCl and NH_3_ at equilibrium with the particle phase. This suppression becomes more pronounced as the aerosol concentrations increase. The specific *a*_w_ used in the model setting was chosen based on HTDMA measurements showing that for both Na_2_SO_4_ and the prepared salt mixture, the point of crystallization is at *a*_w_ ∼0.4 to 0.5 (Figure 2). For comparison, we also model the effect at *a*_w_ 0.30.

Comparing the measured (Figure 4a) and modeled results (Figure 4b), we note that even if the concentration dependence of the ion ratio shows the same principle and is in good agreement, there are some deviations in the absolute values. Such deviations are expected due to experimental uncertainties. In this work, we do not attempt to quantify the losses to the gas phase but simply show that the losses of ions to the gas phase may be significant at normal prevailing laboratory conditions. Examples of experimental uncertainties are the solute mass concentrations at the RH when most evaporation takes place, largely governing the partitioning between the gas phase and particle phase of the solute ions, and the RH gradient in the dryer. Furthermore, the aerosol mass concentration given in the lower *x*-axis of Figure 4a is estimated from the number size distributions measured after the drier, without accounting for the potential loss of particles in the drier or the evaporation of solutes from the particles as we here argue is taking place. Additionally, calculating the particle mass concentration from the SMPS data will include any error related to the sizing or counting of the particles and the errors introduced by assuming spherical particles of a density of 2 g/cm^3^.

## 6 Final Discussion

Laboratory experiments involving nebulization and drying of solutions containing the four most atmospherically abundant ions, Na^+^, Cl^–^, NH_4_^+^, and SO_4_^2–^, can experience considerable losses of solute ions Cl^–^ and NH_4_^+^ to the gas phase through the evaporation of HCl and NH_3_. This evaporation is predicted by thermodynamic models, but only if the aerosol particle mass concentrations are sufficiently low. Since the thermodynamic modeling often is focused on the relative composition of different components, the absolute concentrations are seldom inserted. This can lead to the mass loss to the gas phase not being correctly predicted and the resulting change in the composition of the particle due to the evaporation being overlooked.

The particle mass concentration range where the relative loss to the gas phase becomes significant coincides with the mass concentration range prevalent in many laboratory-based aerosol studies. This means that the chemical properties of the generated particles may be different from those expected based on the nominal experimental settings. The extent of the change in the chemical composition depends on the mass concentration of solutes in air during drying, shifting the partitioning between the gas/particle phase of certain ions (i.e., Cl^–^ and NH_4_^+^). The described phenomenon is also relevant at the particle mass concentrations found in the ambient conditions and should, therefore, also be considered when predicting the chemistry of atmospheric aerosol particles. The phenomenon may hypothetically also introduce artifacts in experimental studies of ambient aerosols. To our knowledge, the direct impacts of these aerosol phenomena on air quality and climate have not been addressed. Considering our planet’s ubiquitous sulfate aerosol, ocean-dominated surface, and the continuous cycling of aerosol through cloud processes (typically including multiple cycles of drying/humidification), it warrants further investigation.

Losses to the gas phase through evaporation of HCl and NH_3_ may be a major explanation for previously observed deviations between laboratory studies and the theory of water uptake of mixtures containing both Cl^–^ and NH_4_^+^, with or without organic components. For mixed inorganic–organic aerosols, such deviations are often attributed to the organic fraction based on the assumption that the inorganic fraction is well understood. This thermodynamic behavior of inorganic droplet solutions is well established in theory but often overlooked, resulting in pronounced errors in the interpretation of experimental data such as particle water uptake. These errors may introduce inaccurate data to explanatory models that may propagate to air quality or climate models.

## Supporting Information Available

Additional hypotheses tested to explain the deviations between theory and experimental results found in the paper by Svenningsson and co-authors^33^ (Supporting Information 1) together with a discussion of results for the mixture representing sea salt particles (MIXSEA); effect of two different assumptions of solution droplet density on the HGF (Supporting Information 2); model results of evaporation with varying NaCl to (NH_4_)_2_SO_4_ ratios (Supporting Information 3); and mass spectra from the AMS (Supporting Information 4) (PDF)
