# Correction to “Missed Evaporation from Atmospherically Relevant Inorganic Mixtures Confounds Experimental Aerosol Studies”

**DOI:** 10.1021/acs.est.3c02233

**Published:** 2023-04-21

**Authors:** Jenny Rissler, Calle Preger, Axel C. Eriksson, Jack J. Lin, Nønne L. Prisle, Birgitta Svenningsson

The corrected Acknowledgments are as follows.

B.S. acknowledges SRA MERGE for creating a stimulating scientific community. B.S. and J.R. acknowledge FORMAS for financial support. C.P. acknowledges the Lund Institute of Technology for postdoc funding. J.R. and A.C.E. further acknowledge financial support via Grant AEROMET II 19ENV08. N.L.P. and J.J.L. acknowledge funding from the Academy of Finland (Grants 257411, 308238, 314175, 331532, and 335649) and the European Research Council (ERC) under the European Union’s Horizon 2020 Research and Innovation program (Project SURFACE, Grant Agreement 717022).

