# Supplementary material for: Missed Evaporation from Atmospherically Relevant Inorganic Mixtures Confounds Experimental Aerosol Studies
Source: Environ Sci Technol. 2023 Feb 9;57(7):2706–14. doi: 10.1021/acs.est.2c06545 (PMC9948290; doi:10.1021/acs.est.2c06545)
Supplement: Supplementary file 1 — es2c06545_si_001.pdf [file es2c06545_si_001.pdf]

Supplemental Information

Missed evaporation from atmospherically relevant inorganic mixtures confound experimental aerosol studies

Jenny Rissler*, Calle Preger, Axel C. Eriksson, Jack J. Lin, Nønne L. Prisle*, Birgitta Svenningsson*

Contents

Supplemental Information 1, page S2-S4

Table S1

Figure S1

Supplemental Information 2, page S5
Figure S2

Supplemental Information 3, page S6
Figure S3

Supplemental Information 4, page S7

Figure S4

References, page S7

Supplemental Information 1

In the study by Svenningsson et al. (2005), the measured hygroscopic growth factors (HGFs) of particles composed by a mixture representing sea spray, MIXSEA (based on Raes et al. 2000), was significantly lower than the HGF estimated using the Zdanovski-Stokes-Robinson (ZSR) method (Stokes and Robinson, 1966). In the ZSR method, the HGFs of the mixtures are based on the HGFs of the pure compounds at a given water activity (Stokes and Robinson, 1966), assuming volume additivity. MIXSEA is a mixture of NaCl, $(NH_4)_2SO_4$, Succinic acid ($C_4O_4H_6$) and Fulvic acid ($C_{33}H_{33}O_{19}$).

The deviation between the measured HGF and those resulting from the ZSR mixing rule could potentially be explained by a strong surface to bulk partitioning of the surface-active organic compounds, resulting in fewer soluble entities in the bulk. Another hypothesis that can explain the observation is a strong interaction between the organic and inorganic components.

HTDMA measurements were also performed also for a mixture of only the inorganic components of MIXSEA: a mixture of NaCl and $(NH_4)_2SO_4$ prepared in molar ratios 2:1 so that the molar ratio of the ions present (Na^+, Cl^-, NH_4^+, SO_4^{2-}) becomes 1:1:1:0.5 (INORGMIX). The MIXINORG also showed considerable deviations between the measured HGF and those predicted by the ZSR mixing rule. However, for this mixture the main difference was found in the shape of the hygroscopic growth curve and a pronounced shift in point of deliquescent (shown in Figure 2). The mass, volume, and mole fractions of the pure compounds in the mixtures is given in table S1 together with chemical formulas, densities of the compounds used in the calculations.

In the original study by Svenningsson and co-authors, the ZSR-model calculations were made assuming using the HGF of the ingoing salts and organic compounds were the same as those formed after nebulizing and drying the particles. When applying thermodynamic models such as the E-AIM-model, it becomes evident that after dissolving the compounds, nebulizing, and drying, preferentially Na_2SO_4 and NH_4Cl are formed to the most possible extent rather than NaCl and $(NH_4)_2SO_4$. Redoing the ZSR-calculations instead using a mixture of Na_2SO_4, NH_4Cl, $(NH_4)_2SO_4$ (and for MIXSEA also Fulvic acid and Succinic acid), however, result in very similar HGFs as the previous assumption of compounds formed.

Other explanations for that the measured HGF of MIXSEA is lower than expected from the ZSR mixing rule could be that the dry particle selected by the first DMA in the HTDMA contain a lower number of soluble entities than assumed based on the mobility diameter – possibly explained by either that *i*) the dry density is lower than assumed, or *ii*) that crystal water (hydrates) is being present. A third explanation leading to that the dry particle of a certain mobility diameter contains less soluble entities is *iii*) non-spherical particles (dynamic shape factor of >1).

Another potential explanation identified for the deviation between predicted and measured HGFs is that there are considerable losses to the gas phase, namely by the evaporation of HCl(g) and NH_3(g), which is the main hypothesis that in the end provided the best explanation of the observations and elaborated in the main text.

To explore the hypotheses the ZSR mixing rule was applied using:

(i) HGF-data data from HTDMA of the pure compounds (when available)
(ii) HGF data from EDB for the pure compounds (e.g. for the inorganic salts since no EDB data was available for the organics)
(iii) The same assumptions as in (ii) but using a lower density for $(NH_4)_2SO_4$ of 1730 kg/m3 (equivalent to a dynamic shape factor of 1.26 if assuming the tabulated bulk density of $(NH_4)_2SO_4$ without crystal water)

(iv) The same as in (i-iii) assuming full evaporation of HCl(g) and NH_3(g) in equivalent molar ratios

The origins of the EDB-data are presented in Table 1. Note that fulvic acid is not a single well-defined chemical compound and the density and molecular weight are estimated averages for the employed reference material (Averett et al., 1989). No EDB-data was found for Fulvic and Succinic acid. For Fulvic acid HGF from HTDMA data was used in all models. Since the point of deliquescence for succinic acid is ~98% RH (the very highest RH in the HTDMA data), the HGF of Succinic acid was estimated using a van't Hoff factor of 1 (assuming ideal behavior). In the calculations of the HGF of the mixture, the solubility (88 g/L) was used, leading to a successive dissolution. It should however be noted that Fulvic and Succinic acid only contributed to a relatively small extent to the water uptake.

Table S1. Description of the mixtures under the assumptions used calculating HGF by the ZSR-model. The values given in the three last columns are volume fraction·100 (%). The values in brackets are the volume fractions using the low density for Na_2SO_4 (1730 kg/m3). Values in parenthesis are the equivalent mole fractions (does not vary with assumed density).

		ρ (kg/m3)	EDB data reference	Composition of prepared mixture (%)	Composition after spraying and drying (%) (i), (ii), and (iii)	Composition assuming evaporation (%) (iv), (v), and (vi)
MIXSEA	NaCl	2170	-	25 (52)	-	-
	$(NH_4)_2SO_4$	1770	Tang and Munkelwitz, 1994	51 (38)	12 [15] (17)	25 [21] (26)
	Na_2SO_4	2660 [1730[2)]]	Cohen et al., 1987	-	26 [34] (25)	38 [49] (54)
	NH_4Cl	1527	Cohen et al., 1987	-	33 [29] (52)	-
	Fulvic acid[1)] ($C_{33}H_{33}O_{19}$)	1500	NA	12 (1.4)	12 [11] (1.4)	19 [15] (2.9)
	Succinic acid ($C_4O_4H_6$)	1572	NA	12 (8.6)	12 [10] (8.6)	18 [15] (18)
INORGMIX	NaCl	2170	-	42 (67)	-	-
	$(NH_4)_2SO_4$	1770	-	58 (33)	-	-
	Na_2SO_4	2660 [1730[2)]]	Cohen et al., 1987	-	43 [54] (33)	100 [100] (100)
	NH_4Cl	1527	Cohen et al., 1987	-	57 [46] (67)	-

[1)] Suwannee River Reference Fulvic Acid is in itself a mixture of many unknown substances, and it is represented by an estimated average composition (Averett et al., 1989).

[2)] The density of Na_2SO_4 of 1730 g/cm3 results from fitting the particle effective density of the particles needed to achieve the same HGFs measured by the HTDMA of pure Na2SO4 as those predicted from measured water activity by EDB, as explained in the result section. The density was fitted from HGF in the interval 80-95% RH.

The resulting HGFs based on the water uptake of each individual inorganic salt, either from HTDMA data (i) or EDB data (ii), are shown in the left upper panel of Figure S1 (S1a) together with the measured HGFs of MIXSEA. In Figure S1a is also shown HGFs estimated from the ZSR-model, but assuming a density of Na_2SO_4 of 1730 g/cm3 in the estimation of HGFs from EDB data. The reason for using the lower density of Na_2SO_4 is an observed deviation in the HGF measured by the HTMDA and that based in EDB-data (Cohen et al, 1987) of the pure salt, with lower HGF measured by the HTDMA than when estimated from EDB-data, likely explained by porous non-spherical particles and/or crystal water. In the literature one forms of Na_2SO_4 of lower density have been reported (glaube salt, 1740 g/cm^3). The equivalent graph for MIXINORG is shown in Figure S1c.

In the main text of the paper, we present the hypothesis that the difference between measured and modeled HGFs are due to the evaporation of HCl(g) and NH_3(g) in equivalent molar ratios, occurring during the drying phase of the droplets after nebulizing the solutions in the laboratory. To try the main hypothesis of evaporation, the HGF was modeled under the assumption that equal amounts of HCl and NH_3 evaporate from the particle. The results are shown in the upper right panel of Figure S1 (S1b) for MIXSEA and lower right panel for MIXINORG (S1d). Assuming a complete evaporation leads to a decrease in hygroscopicity for MXSEA mainly because the organic fraction increases as inorganics evaporate, since the organics typically have lower HGFs than the inorganic salts. The effect in the absolute HGFs for MIXINORG is not as pronounced. For this mixture, the main difference is in the shape of the hygroscopic growth curve and the obvious shift in point of deliquescent, further evaluated as a proof of the hypothesis in the main text.

The measured and modeled HGFs is in good agreement assuming loss of all NH_4Cl(s) to the gas phase by evaporation of HCl(g) and NH_3(g) for both MIXSEA and INORGMIX.

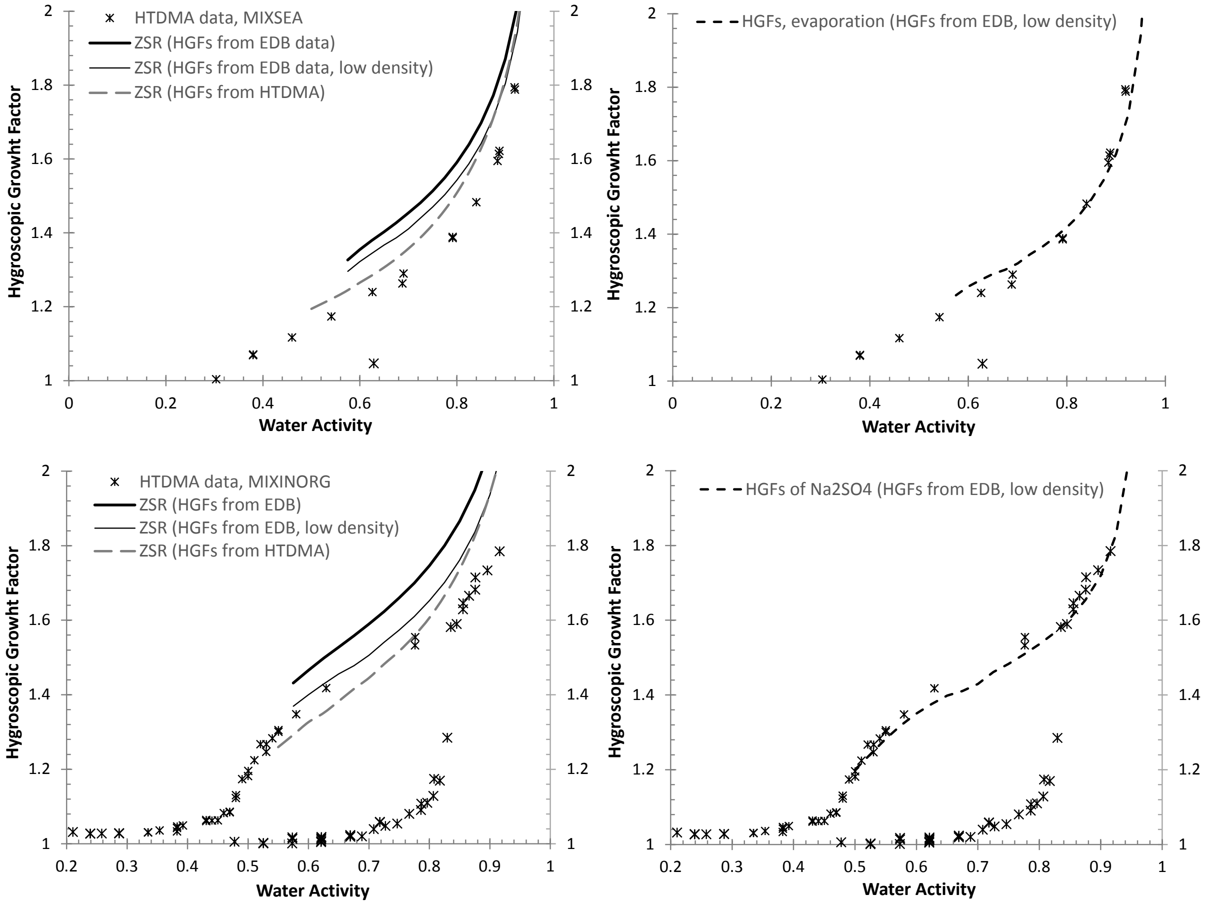

Figure S1. ZSR modeling of the hygroscopic growth factors of the mixtures based on various data and assumptions: i) ZSR based on HGF of pure compounds measured by the HTDMA, ii) same as i) but replacing HGF with data from EDB of the inorganic salts, iii) the same assumptions as in (ii) but using a dry density for Na_2SO_4 of 1730 kg/m^3, and iv) same as in (i) and assuming full evaporation of HCl(g) and NH_3(g) (right panel). For INORGMIX, in the lower right panel pure Na_2SO_4(s) corresponds to the assumption of full evaporation of HCl(g) and NH_3(g).

Supplemental information 2

In the paper we are working with a molar ratio of NaCl to $(NH_4)_2SO_4$ of 2:1, i.e. a molar fraction of NaCl (xNaCl) of 2/3. This molar ratio allows for maximum evaporation of a combination of HCl and NH_3. To get a view of how the evaporation takes place for other molar ratios we used the E-AIM model. We used the model corresponding to the settings in case 3 in Table 1, with the sum of NaCl and $(NH_4)_2SO_4$ concentrations being 4 $*10^{-7}$ moles m^{-3}, at a water activity of 0.3. This is chosen as a case with high evaporation (in contrast to a high mass concentration and a high a_w). For xNaCl of 2/3, the evaporation of both HCl and NH_3 is high (figure S2). For higher xNaCl (right part of figure S2), the fraction of Cl^- evaporating decreases (i.e. the aerosol will be dominated by NaCl) and for lower xNaCl (left part of figure S2), most of the HCl will be evaporated, and the evaporating fraction of NH_3 decreases (i.e. the aerosol is dominated by $(NH_4)_2SO_4$).

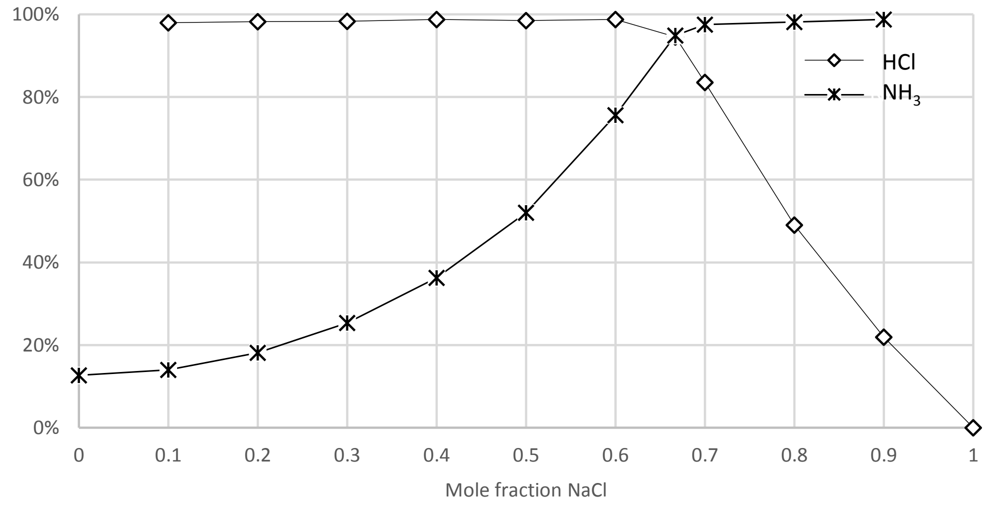

Figure S2. Percent of NH_4^+ and Cl^- lost to gas phase as NH_3 and HCl according to the E-AIM model for a total concentration (NaCl and $(NH_4)_2SO_4$) of 4 * 10^{-7} moles m^{-3} and a water activity of 0.3 as a function of molar fraction of NaCl, xNaCl = [NaCl]/([NaCl + [$(NH_4)_2SO_4$].

Supplemental information 3

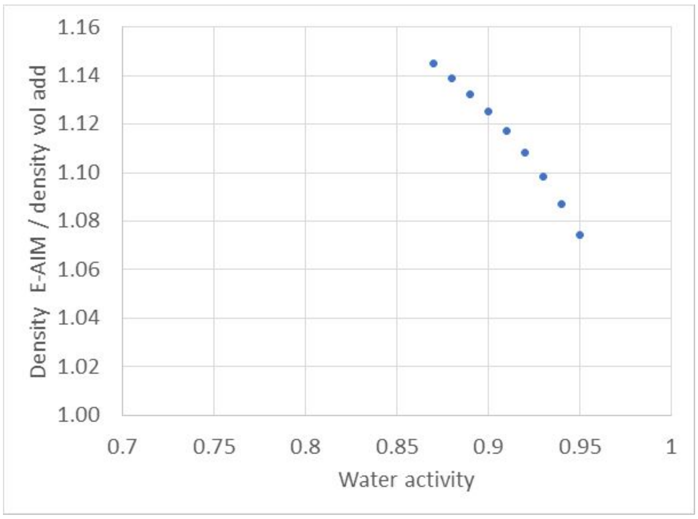

Figure S3. Effect on HGFs of assumed droplet densities in case 2b (vol additivity) and 2c (density according to E-AIM). The effect can only be compared for water activities high enough for all solutes to be dissolved (water activity larger than 0.87).

Supplemental information 4

Figure S3 shows the AMS mass spectra from nebulizing solutions of $(NH_4)_2SO_4$), NaCl and a mixture of $NaCl:(NH_4)_2SO_4$ in 2:1 molar ratio. The mixture produces different spectra than would be obtained from super positioning the pure solution spectra (e.g. the Na containing peaks which are very abundant in the NaCl spectrum are virtually absent in the mixtures).

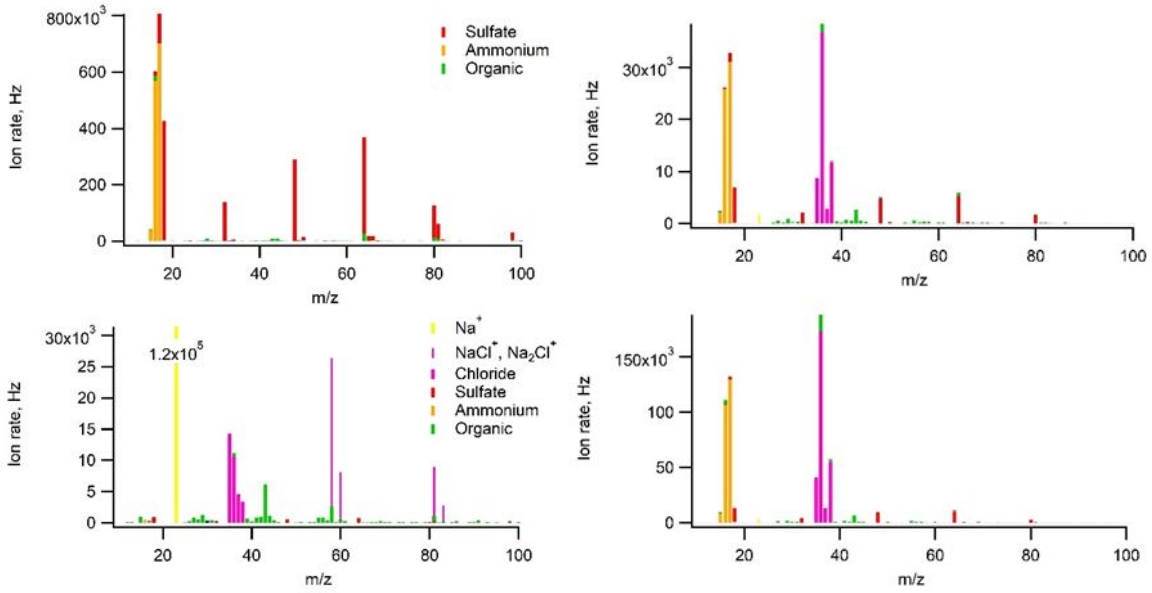

Figure S4. Mass spectra from nebulizing solutions of ammonium sulfate (left, top) sodium chloride (left, bottom) and 2:1 (molar) mixture of $NaCl:(NH_4)_2SO_4$ at low (right, top) and high (right, bottom) concentration.

References

Tang, I. N. (1996) Chemical and size effects of hygroscopic aerosols on light scattering coefficients, J. Geophys. Res., 101, 19245-19250

Tang, I. N. and Munkelwitz, H. R. (1994) Water activities, densities and refractive indices of aqueous sulfates and sodium nitrate droplets of atmospheric importance, solutions , J. Geophys. Res, 99, 18801-18808

Raes, F., van Dingenen, R., Vignati, E., Wilson, J., Putaud, J. P., Seinfeld, J. H., and Adams, P. (2000) Formation and cycling of aerosols in the global troposphere, Atm. Environ., 34, 4215-4240

Stokes, R. H. and Robinson, R. A. (1966) Interactions in aqueous nonelectrolyte solutions: I. Solute-solvent equilibria., J. Phys. Chem., 70, 2126-2130

Averett, R. C., Leenheer, J. A., McKnignt, D. M. , and Thorn, K. A. (1989) Humic substances in the Suwannee River, Georgia: Interactions, properties, and proposed structures, U.S.G.S. report.

Cohen, M.D, Flagan, R.C, and Seinfeld, J.H (1987) Studies of concentrated electrolyte solutions using the electrodynamic balance. 1. Water activities for single-electrolyte solutions, J. Phys. Chem., 91, 17, 4563–4574, https://doi.org/10.1021/j100301a02
